# Speckle-type POZ protein could play a potential inhibitory role in human renal cell carcinoma

**DOI:** 10.1186/s12885-022-10340-w

**Published:** 2022-12-07

**Authors:** Zhi Chen, Zuan Li, Chunlin Li, Bingcai Li, Haojian Wang, Deyong Nong, Ximing Li, Guihai Huang, Junhao Lin, Wei Li

**Affiliations:** Department of Urology, The People’s Hospital of Guangxi Zhuang Autonomous Region, Guangxi Academy Of Medical Sciences, Nanning, China

**Keywords:** Renal cell carcinoma, Speckle-type POZ protein, In vitro proliferation, Migration, Invasion, Apoptosis

## Abstract

**Background:**

Speckle-type POZ protein(SPOP), a substrate adaptor of Cul3 ubiquitin ligase, plays crucial roles in solid neoplasms by promoting the ubiquitination and degradation of substrates. Limited studies have shown that SPOP is overexpressed in human renal cell carcinoma (RCC) tissue. However, the exact role of SPOP in RCC remains unclear and needs to be further elucidated. The present study showed that SPOP was expressed at different levels in different RCC cell lines. The purpose of this study was to explore the roles of SPOP in the biological features of RCC cells and the expression levels of SPOP in human tissue microarray (TMA) and kidney tissues.

**Methods:**

Here, SPOP was overexpressed by lentiviral vector transfection in ACHN and Caki-1 cells, and SPOP was knocked down in Caki-2 cells with similar transfection methods. The transfection efficiency was evaluated by quantitative PCR and western blotting analyses. The role of SPOP in the proliferation, migration, invasion and apoptosis of cell lines was determined by the MTT, wound-healing, transwell and flow cytometry assays. Moreover, the cells were treated with different drug concentrations in proliferation and apoptosis assays to investigate the effect of sunitinib and IFN-α2b on the proliferation and apoptosis of SPOP-overexpressing cells and SPOP-knockdown RCC cells. Finally, immunohistochemical staining of SPOP was performed in kidney tissues and TMAs, which included RCC tissues and corresponding adjacent normal tissues.

**Results:**

Overexpression of SPOP inhibited cell proliferation, migration and invasion and increased cell apoptosis. Interestingly, sunitinib and IFN-α2b at several concentrations increased the proliferation inhibitory rate and total apoptosis rate of cells overexpressing SPOP. The findings of the present study showed that the SPOP protein was significantly expressed at low levels in most clear cell RCC (ccRCC) tissues and at relatively high levels in the majority of adjacent normal tissues and kidney tissues. Kaplan–Meier survival analysis showed that there was no statistically significant difference in cumulative survival based on the data of different SPOP expression levels in TMA and patients.

**Conclusions:**

In contrast to previous studies, our findings demonstrated that overexpression of SPOP might suppress the progression of RCC cells, which was supported by cell experiments and immunohistochemical staining. SPOP could be a potential tumour inhibitor in RCC.

**Supplementary Information:**

The online version contains supplementary material available at 10.1186/s12885-022-10340-w.

## Introduction

Renal cell carcinoma (RCC) ranks as the sixth most frequently diagnosed cancer in men and the 10th most frequently diagnosed cancer in women in the United States. Moreover, it has been reported that RCC incidence and mortality are increasing around the world [1–3]. Clear cell carcinoma represents the most common tissue subtype, accounting for 70% ~ 90% of RCCs. Approximately 25% of patients diagnosed with RCC have evidence of advanced disease or metastases, although the increasing incidence is correlated with the wide use of multiple medical techniques for RCC screening [4, 5]. Primary localized renal cancers can be cured by radical or partial nephrectomy. The cytoreductive nephrectomy (CN) can be performed before systemic treatment for mRCC patients with good physical fitness, relatively normal physical indicators, and resectable primary according to the National Comprehensive Cancer Network,and it could improved overall survival and provided symptomatic benefit in combination with cytokine therapies,but therapeutic options for mRCC patients have remarkably expanded over the last 20 years.Targeted therapy is currently the standard treatment for mRCC [6, 7].The treatment outcome for patients with metastatic RCC (mRCC) has improved since the introduction of VEGF inhibitors and agents targeting the PI3K/Akt/mTOR signalling pathway, including combination strategies, such as lenvatinib and everolimus. Moreover, anti-angiogenic/anti-PD1/L1 combinations (ICIs) have become globally accepted options in the upfront metastatic setting, overall survival has been improved with different ICI-based combination strategies compared to single-agent Sunitinib [7, 8]. Unfortunately, drug resistance is still a major problem and previous systemic treatment options could not provide long-term efficacy for mRCC, which ultimately becomes resistant to first-line drugs [9–11]. The molecular mechanism of resistance to targeted therapy for advanced or metastatic RCC has become a research hotspot.

Speckle-type POZ protein (SPOP), a novel nuclear speckle-type protein, was first identified in 1997 [12]. Previous studies have shown that SPOP, as a substrate adaptor of cullin3-RING ubiquitin ligase (CRL3), recruits substrates to CRL3 for ubiquitination and degradation, such as the androgen receptor (AR), steroid receptor coactivator-3(SRC-3) and PTEN proteins [13–15]. Over the past decades, SPOP has been confirmed as a tumour suppressor in several cancers, including prostate cancer, lung cancer and gastric cancer, and studies of the differential expression levels and mutation status of SPOP have indicated that SPOP plays different roles in cancer cell development [16]. It is well-known that dramatically decreased SPOP expression is negatively correlated with tumorigenesis in gastric cancer tissues [17]. In addition, SPOP gene mutation is the most common missense point mutation in prostate cancer and affects the progression of prostate tumours through coordinated regulation of the PI3K/mTOR and AR signalling pathways [18, 19]. Interestingly, to date, mutations in SPOP have not been found in RCC tumours [14, 19]. Some studies have shown overexpression of SPOP in the cytoplasm of clear cell RCC (ccRCC) cells and have indicated some correlation with high pathological stages, lymph node invasion and metastasis [20, 21]. However, the exact role of SPOP in the biological features of RCC and its potential molecular mechanism in RCC tumours remain unclear. Aim of the present study was to explore the roles of SPOP in the biological features of RCC cells and the expression levels of SPOP in human RCC and kidney tissues.

## Materials and methods

### Cell lines and cell culture

The ACHN and Caki-1 cell lines were obtained from the Cell Bank of Type Culture Collection of the Chinese Academy of Sciences. Caki-2 cell lines were provided by Guangzhou Cell cook Biotech Co. Ltd. ACHN, Caki-1 and Caki-2 cell lines were maintained in Dulbecco's modified Eagle's medium (DMEM) (Gibco, shanghai,china) supplemented with 10% foetal bovine serum (FBS, 10,270–106, Gibco, shanghai,china). All the cells were cultured in a humidified incubator containing 5% CO_2_ at 37 °C and were used in further experiments.

### Transfections

To explore the effect of SPOP on the biological features of RCC, cells with different levels of SPOP protein expression were constructed by the lentiviral vector transfection.The plasmids were transfected into cells with Lipofectamine 2000 according to the manufacturer’s instructions. The following day, the cells were cultured with media containing neomycin and selected for two weeks to obtain stably transfected cells. The SPOP plasmid or shRNA plasmid was packaged and transfected into retroviral packaging cells. Retroviral supernatants were added to the cells, spun for 45 min at 1800 rpm and incubated for 4 h at 37 **℃**. The cell medium was switched to medium supplemented with puromycin for one week to select stable cell lines. The overexpression and knockdown efficiency of SPOP in the cells was tested by western blot and qPCR analyses.

### Western blot analysis

The expression of SPOP in the cells was tested by western blot. As previously described [22], cell protein samples were harvested using RIPA buffer, and 20 μg of the protein sample was separated on 12% SDS–polyacrylamide gels followed by wet transfer at room temperature. The blots were then blocked with non-fat milk, followed by incubation with diluted primary antibody overnight at 4 °C. The membranes were washed three times with TBST for 5 min and subsequently incubated with the appropriate secondary antibody conjugated to IRDye800 at room temperature for 2 h. Protein bands were visualized with the ECL detection system and analysed using the ImageJ software.

### Quantitative real-time PCR analysis

The overexpression and knockdown efficiency of SPOP were verified by qPCR analyses. Total RNA from cells was extracted and reverse transcribed using a cDNA synthesis kit (Invitrogen). Real-time PCR analysis was performed using a LightCycler 96 (Roche, shanghai,china). The peak of the melting curve was defined as the criterion for amplification specificity. The relative expression levels of mRNAs were determined by normalization to the expression levels of the internal control gene GAPDH, and the data were analysed by the ΔΔCt method.

### Cell invasion assay

The capacity of cell invasion was evaluated by the Transwell assay. A total of 2 × 10^5^ cells in serum-free medium were plated on top of the Transwell chamber, which was coated with Matrigel matrix (Corning 354,230, shanghai,china). Medium supplemented with 10% FBS as the chemoattractant was added to the bottom of the chamber. The cells were then incubated in Transwell plates at 37 °C in 5% CO_2_ for 48 h. The non-invading cells at the top of the chamber were carefully removed with a cotton swab. The cells on the lower surface of the Transwell chamber were stained with crystal violet for 30 min after fixation with paraformaldehyde. The inserts were washed three times with PBS(P1020-500, Solarbio,shanghai,china), and the number of invading cells was counted under a microscope(OLYMPUS CKX53, Japan).

### Cell migration assay

Cell migration was determined by the wound-healing assay. A total of 1.2 × 10^5^ cells were plated in a 12-well plate at 37 °C and 5% CO_2_ overnight. A horizontal scratch was then made in the plate using a sterile pipette tip, followed by washing with PBS three times to remove the floating cells. Finally, the cells were incubated in serum-containing medium at 37 °C in 5% CO_2_ for 24 h. The scratch migration area was calculated using the ImageJ software after 0 h and 24 h.

### Cell proliferation and apoptosis assays

For the proliferation assay, 1.5 × 10^5^ cells were cultured in 96-well plates with regular medium at 37 °C and 5% CO_2_ for 24 h. The next day, the culture medium was replaced with medium supplemented with IFN-α2b (20, 80, 4000 and 5000 IU/ml) or sunitinib (2.50, 5.01, 7.00 and 10.05 µmol/L) and incubated for 48 h. During culture, 10 ml CCK-8 chromogenic agent and 100 ml DMEM without FBS were added to the wells and incubated for 1 h. The absorbance (A) at 450 nm was analysed using a microplate reader. The inhibitory rate of cell growth (%) was quantified as follows: (1-(A_treated_)/(A_control_)) × 100%. For the apoptosis assay, 1 × 10^5^ cells were seeded into 6-well plates, allowed to attach overnight and then treated with 10% FBS (control), IFN-α2b (20, 40 and 80 IU/ml), or sunitinib (5.0, 5.5 and 6.0 µmol/L) for 48 h. The cells were collected by centrifugation at 1200 rpm for 5 min at 37 °C. The collected cells were washed with PBS and 50 µl 1 × binding buffer. Then, the cells were stained with Annexin V-APC and 7-AAD and incubated at room temperature in the dark for 15 min. Subsequently, 50 µl 1 × binding buffer was added, and the samples were tested using an Accuri^TM^C6 PLUS flow cytometer (BD Biosciences,USA). The sum of early apoptosis and late apoptosis was defined as total apoptosis.

### Tissue microarrays (TMA) and normal kidney tissues

TMAs of formalin-fixed paraffin-embedded human renal tumour and adjacent normal tissues from the Shanghai Outdo Biotech Company were evaluated. The TMA comprised 180 tissue samples (90 tumour tissue samples and 90 adjacent normal tissue samples) collected from patients who underwent nephrectomy between 2006 and 2008. The 90 RCC specimens included clear cell renal cells (*n* = 88) and papillary cells (*n* = 2). In addition, normal kidney tissues (*n* = 10) obtained from The People’s Hospital of Guangxi Zhuang Autonomous Region were used. Related clinical data, including follow-up time, sex, and tumour stage, were recorded in detail for all patients who signed the informed consent form.

### Immunohistochemical staining

Immunohistochemical staining of the SPOP protein in the cytoplasm of TMA and kidney tissues was performed with appropriate antibodies according to the methods of a previous study [23]. Briefly, paraffin-embedded sections were subjected to deparaffinization, rehydration, and heat-induced antigen retrieval. The sections were incubated with primary SPOP antibody overnight at 4 °C after blocking endogenous peroxidase activity with 3% hydrogen peroxide. Rabbit IgG antibody was used for the isotype control. 3,3'-Diaminobenzidine (DAB) was added as a chromogen followed by counterstaining with haematoxylin. The staining intensity and positive staining rate were assessed by two independent pathologists according to the histologic scoring system (H-score). SPOP expression was scored comprehensively based on the positive staining rate and staining intensity. The positive staining rate was scored as follows: 0 (negative), 1 + (1–25%), 2 + (26–50%), 3 + (51–75%), and 4 + (76–100%). The intensity of cytoplasmic staining was classified as follows: 0 (negative), 1 + (weak), 2 + (moderate), and 3 + (strong). The above two scores were multiplied to obtain the final score. A total score of SPOP immunohistochemical staining ≥ 6 was defined as high expression; otherwise, it was considered low expression.

### Statistical analysis

Statistical analyses and figure preparation were performed using the SPSS 24.0 (SPSS, Inc., Chicago, IL, USA) and GraphPad Prism 7.0 (San Diego, California, USA) software. Values of the in vitro cell experiments are presented as the mean ± standard deviation based on results obtained from at least three independent experiments. Comparisons were made between homogeneous experimental groups using the t-test or ANOVA, as appropriate. The Mann–Whitney U test was used to analyse the differences in SPOP expression between tumour tissues and adjacent normal tissues. Survival analysis was determined by the Kaplan–Meier method and compared by the log rank test. A *p*-value less than 0.05 was considered to be statistically significant.

## Results

### Overexpression of SPOP inhibits the invasion and migration of RCC cells in vitro

**Fig. 1:**
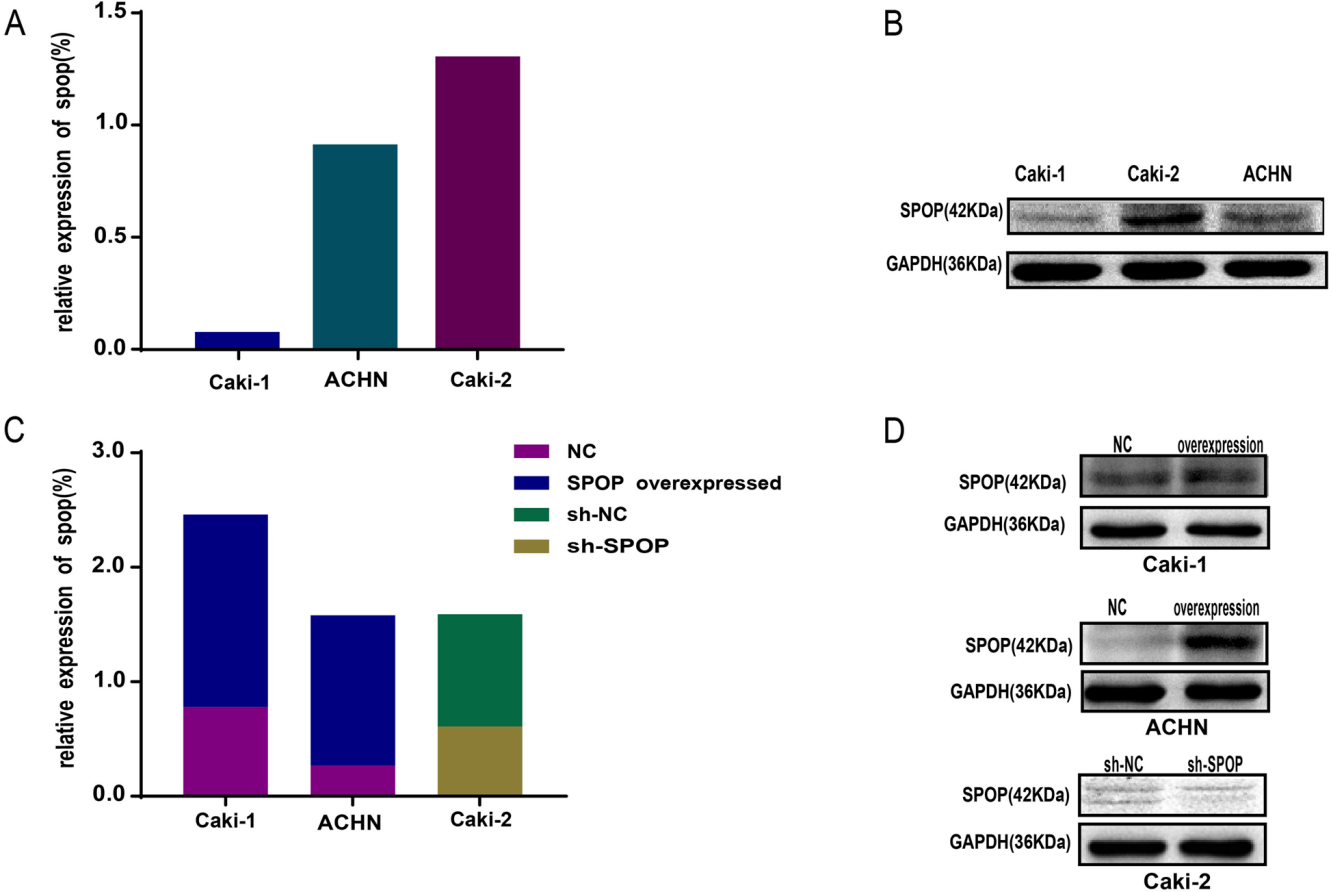
Expression of SPOP in ACHN, Caki-1 and Caki-2 cells. **A** and **B** Relative expression of SPOP in RCC cell lines was measured by western blot analysis. SPOP expression in Caki-2 cells was highest among the examined RCC cell lines. **C** and **D** SPOP was knocked down in Caki-2 cells and upregulated in ACHN and Caki-1 cells through transfection with a lentiviral vector for further experiments. The levels of SPOP in RCC cell lines were determined by western blot. ACHN and Caki-1 cells were divided into the NC group and SPOP-overexpressing group. Caki-2 cells were divided into the sh-NC and sh-SPOP groups

**Fig. 2:**
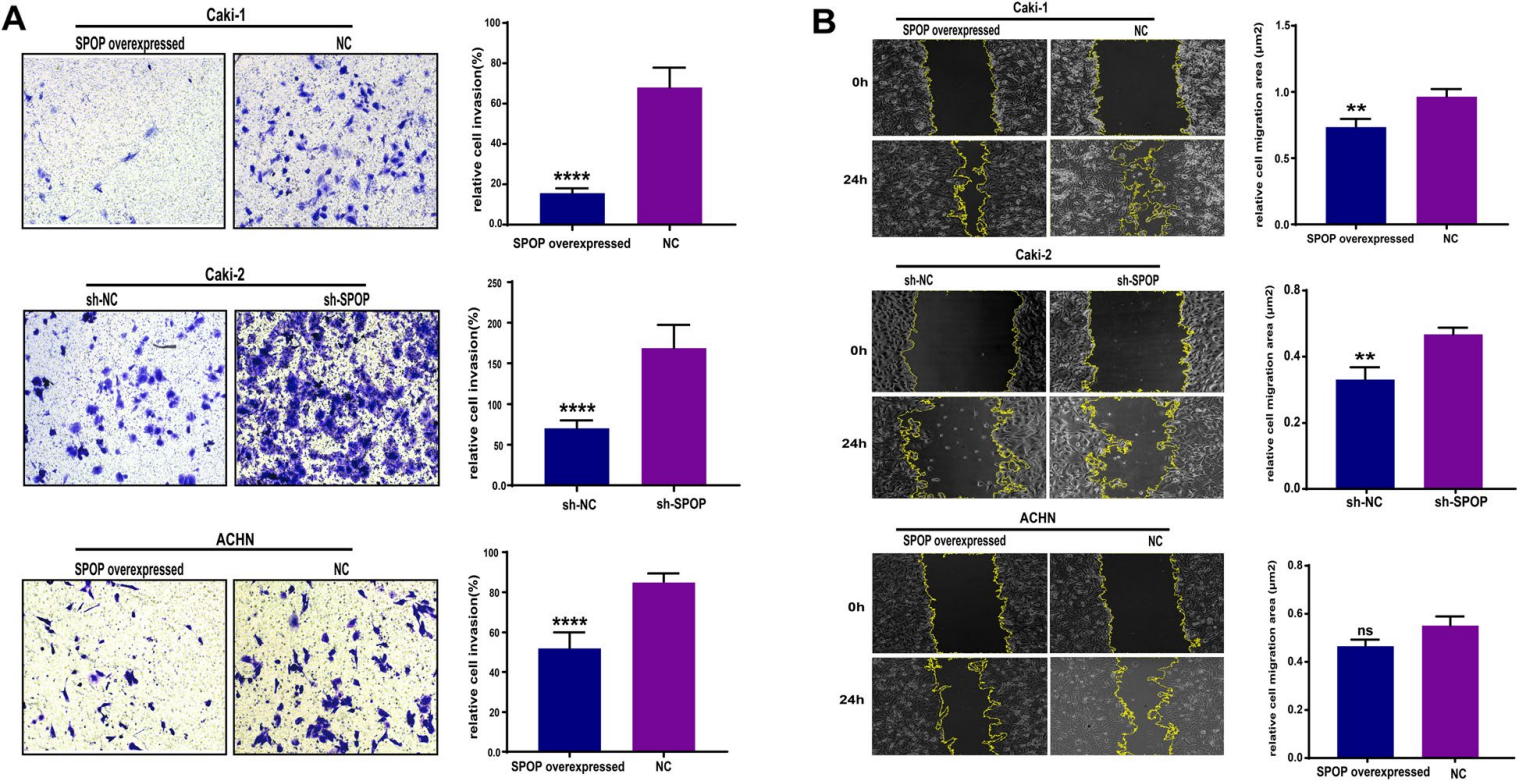
The effect of SPOP expression on the invasion and migration of RCC cells. (**A**) Five high-power fields were randomly selected to count the number of invading cells under a microscope after incubation in transwell inserts for 48 h. Cell invasion was significantly decreased in the SPOP high expression group compared with the SPOP low expression group (****, *P* < 0.0001). (**B**) Cell migration was determined by the wound-healing assay, and the scratch migration area was calculated using the ImageJ software after 0 h and 24 h. Overexpression of SPOP significantly inhibited the invasion ability of Caki-1 and Caki-2 cells (**, *P* < 0.01), and a trend towards a statistically significant difference was observed in ACHN cells. (P = 0.0649, ns)

ACHN, Caki-1 and Caki-2 cell lines are commonly used in RCC studies. Our preliminary western blotting results showed that SPOP expression was significantly downregulated in the ACHN and Caki-1 cell lines and upregulated in Caki-2 cells. (Fig. 1 A and B). To explore the effect of SPOP on the biological features of RCC cells, ACHN and Caki-1 cells were transfected using a lentiviral vector overexpressing SPOP, and SPOP in Caki-2 cells was knocked down with similar transfections using a small hairpin RNA (shRNA) lentiviral vector. The overexpression and knockdown efficiency of SPOP were verified by western blot (Fig. 1 C and D) and (see supplementary data) qPCR analyses. By transwell assays, we found that overexpression of SPOP in ACHN and Caki-1 cells significantly inhibited the invasive ability of the cells after incubation for 48 h compared with the negative control (NC). Similar results were observed in the sh-NC group of Caki-2 cells compared with the SPOP-silenced group (*P* < 0.0001, Fig. 2 A). Moreover, cell migration is another malignant behaviour of cancer cells. The effect of SPOP on cell migration was measured by the wound-healing assay. High expression of SPOP in Caki-1 and Caki-2 cells significantly decreased the migration capacity of RCC cells after 24 h compared to that of cells with low expression of SPOP (*P* < 0.01 Fig. 2 B). A similar phenomenon was observed in ACHN cells; however, no significant difference was noted (P = 0.0649). The above findings suggested that SPOP may play an important role in suppressing the malignant biological behaviour of RCC cells.

### SPOP suppresses RCC cell proliferation and induces cellular apoptosis

**Fig. 3:**
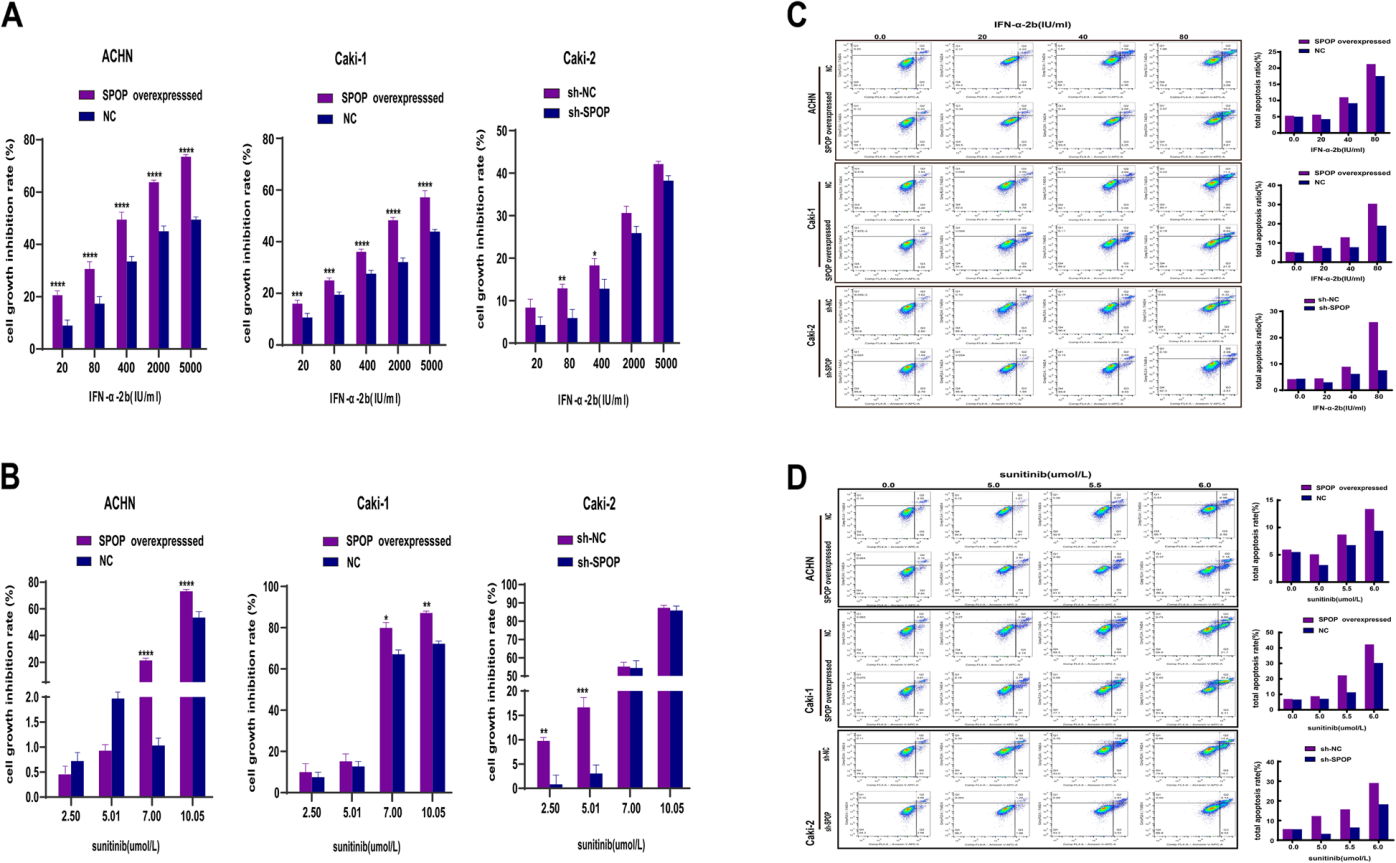
The effects of upregulation or knockdown of SPOP under treatment with sunitinib or IFN-α2b on RCC cell proliferation and apoptosis. (**A** and **B**) The cells were incubated with the indicated drug concentrations for 48 h and subjected to ELISA to analyse the OD450 and calculate the proliferation inhibitory rate of the cells. Sunitinib or IFN-α2b at several concentrations significantly increased the proliferation rate of RCC cells overexpressing SPOP compared to that of RCC cells with low expression of SPOP (*, *P* < 0.05, **, *P* < 0.01. ***, *P* < 0. 001. ****, *P* < 0.0001). (**C** and **D**) RCC cells were treated with different concentrations of sunitinib or IFN-α2b, collected and stained with Annexin V-APC and 7-AAD. The total apoptosis rate consisted of early apoptosis (Q3) and late apoptosis (Q2) and was quantified by flow cytometry. The total apoptosis rate of RCC cells overexpressing SPOP increased with increasing drug concentrations, and the SPOP protein may improve the susceptibility of RCC cells to drug treatments

The data showed that SPOP plays a key role in the invasion and migration of RCC cells. Next, we performed experiments to investigate the effect of SPOP on the proliferation and apoptosis of RCC cells using the MTT assay and flow cytometry. Previous studies suggested that the motility of Caki-2 cells was stronger than that of ACHN and Caki-1 cells, which could be suppressed by IFN or sorafenib, especially when the two drugs were combined, which indicated that Caki-2 cells were more aggressive than ACHN and Caki-1 cells [24]. Advanced or metastatic RCC cannot be cured due to drug resistance, which remains one of the most challenging issues. In the present study, we investigated the effects of first-line drugs (sunitinib and IFN-α2b) on the above cell lines with different SPOP expression levels. The results showed that overexpression of SPOP decreased RCC cell proliferation (Fig. 3 A and B) and induced cellular apoptosis under several drug concentrations (Fig. 3 C and D) compared to low SPOP expression. All of these findings suggested that the SPOP protein may improve the susceptibility of RCC cells to drug treatments.

### SPOP is mainly expressed at low levels in the cytoplasm of ccRCC tissue

**Fig. 4:**
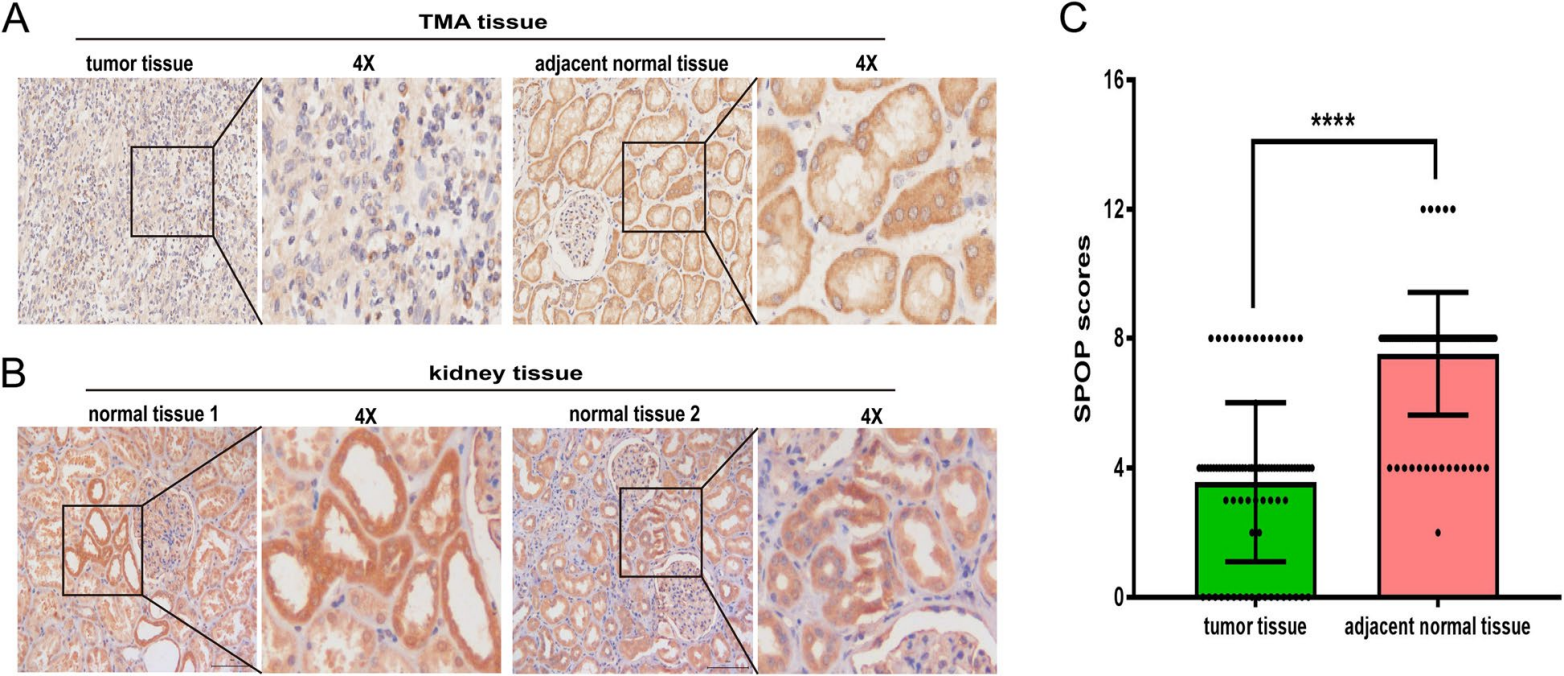
Immunohistochemical staining of SPOP in the cytoplasm of RCC tissues and adjacent normal tissues. SPOP expression in the TMA was assessed by the Aperio image software, and the images were captured at 4X magnification. (**A**) In the TMA, SPOP was significantly expressed at low levels in the cytoplasm of ccRCC tissues and at high levels in adjacent normal tissues. (**B**)Similar results were observed in normal kidney tissues overexpressing the SPOP protein under a 4X field microscope. (**C**) Scores of SPOP expression in TMA are shown as scatter plot with bar (mean with SD) (****, *P* < 0.0001)

Cell experiments indicated that SPOP expression significantly inhibited the growth and progression of RCC cells. To determine SPOP expression in human RCC tissues and normal kidney samples, immunohistochemical staining was performed on TMA tissues consisting of ccRCC tissues (*n* = 88), papillary RCC tissues (*n* = 2) and corresponding adjacent normal tissues. We found that the expression of SPOP protein in the cytoplasm was significantly downregulated in 83% of ccRCC tumour tissues and upregulated in 88% of adjacent nontumor tissues (*P* < 0.0001, Fig. 4 A and C). To further confirm these preliminary results, we continued to analyse SPOP expression in the normal kidney samples. Consistent findings were observed in the immunohistochemical staining of normal kidney tissues (Fig. 4 B), in which the SPOP protein was mainly overexpressed in the cytoplasm of kidney tissues. This is quite different from previous studies, which showed that the SPOP protein was overexpressed in greater than 80% of RCC tissues, even in nearly 100% of primary ccRCCs showing SPOP accumulation, and negative in 82% of normal kidney tissues [20, 21, 25].

### SPOP protein expression and clinical correlations in RCC

**Fig. 5:**
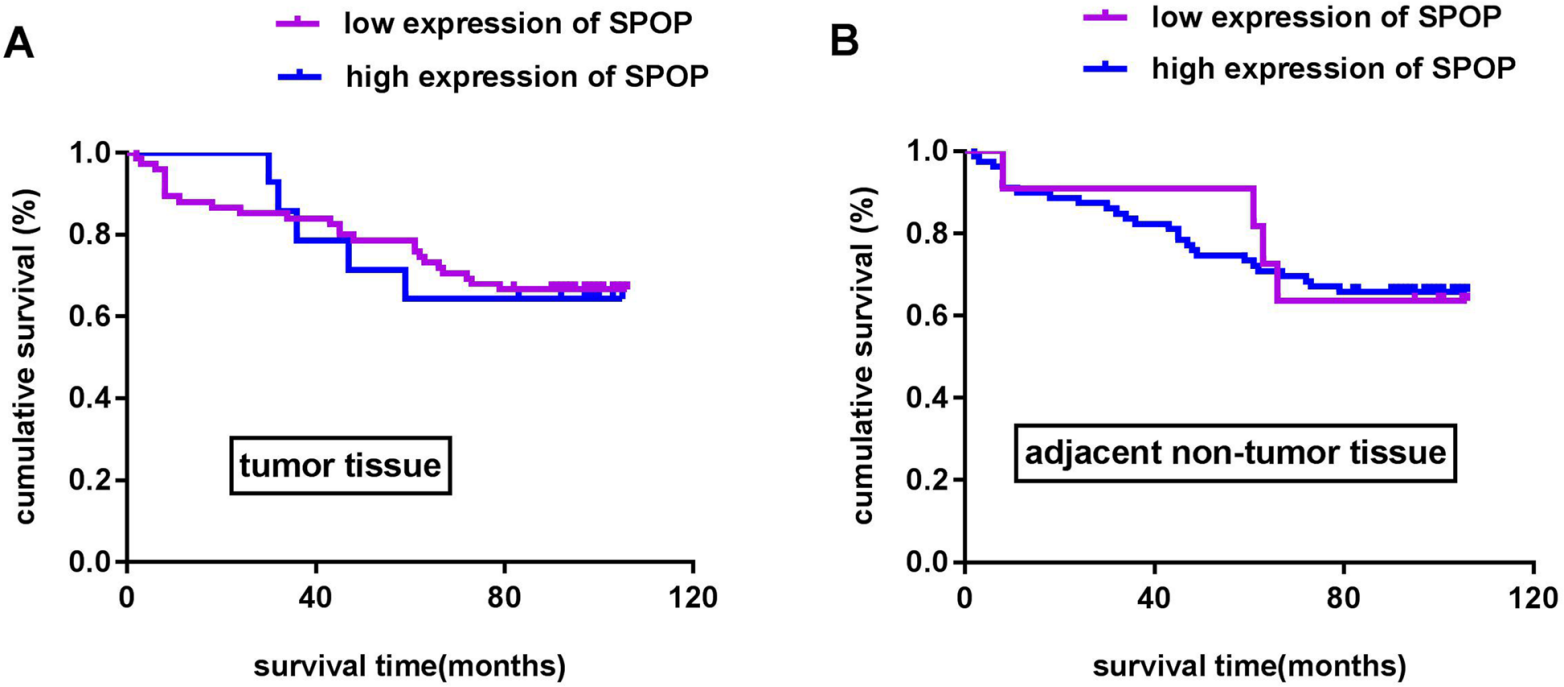
Kaplan–Meier survival analysis. (**A**) No statistically significant survival difference was noted between patients with high or low expression of SPOP in tumour tissues (*P* > 0.05). (**B**) There was no statistically significant survival difference between patients with different expression levels of SPOP in adjacent nontumor tissues (*P* > 0.05)

Based on the immunohistochemical results, we showed that the SPOP protein was expressed at lower levels in most RCC tissues. Next, we investigated whether SPOP expression was associated with overall survival. Survival analysis was performed using the Kaplan–Meier method based on SPOP expression levels and follow-up time. We found that there was no significant correlation between SPOP expression and patient cumulative survival (*P* > 0.05), as shown in Fig. 5. Analysis of the Kaplan–Meier curves suggested that the role of SPOP in RCC remains controversial, and the mechanism of action of SPOP needs to be explored further. However, all of our findings suggest that the SPOP protein could act as a protective factor in RCC.

## Discussion

SPOP, a CRL3 substrate adaptor protein, plays an important role in the development of some cancers [13–15]. Over the past decade, the potential functions of SPOP in urologic cancers have gradually attracted much attention from investigators. SPOP was found to be the most common missense mutated gene in human prostate cancers and has been shown to be associated with the pathogenesis of primary prostate tumours, but SPOP mutations in RCC tumours have not yet been reported [18, 19, 26, 27]. Recent studies have shown that SPOP is an oncoprotein that is overexpressed in RCC [20, 21, 28]. However, the results of our preliminary cell experiments showed that overexpression of SPOP inhibited RCC cell proliferation, migration and invasion and increased cellular apoptosis rates. Similar to our cell experiment results, some studies also found that high expression of SPOP suppressed the malignant biological behaviour of cancer cells in vitro via ubiquitin-dependent proteolysis of the signalling pathway [17, 29–31]. Therefore, SPOP may be associated with inhibition of the aggressiveness of RCC cells.

SPOP plays key roles in cancer development by promoting ubiquitination and degradation of the substrate protein of specific signalling pathways. For example, SPOP has a definitive tumour suppressing role in gastric cancer by promoting the degradation of the transcription factor Gli2 of the Hedgehog (Hh)/Gli2 signalling pathway [17]. In an in vitro drug sensitivity experiment, we found that the proliferation inhibitory rates of cells were significantly increased and cellular apoptosis was induced when SPOP was overexpressed in RCC cells that were treated with sunitinib or IFN-α2b. Sunitinib, a tyrosine kinase inhibitor targeting the VEGF receptor, has been the first-line targeted therapy for patients with mRCC who have been classified as having MSKCC intermediate-risk or poor-risk disease and has shown an improvement in survival [9, 32]. Cellular migration, proliferation and survival of cancer cells as well as endothelial cell differentiation are mainly driven by VEGF/VEGFR activation, which in turn activates the PI3K/Akt/mTOR signalling pathway [33, 34]. One study showed that 4-chloro fascaplysin, a marine sponge alkaloid derivative, inhibited tumour growth and VEGF-mediated angiogenesis by disrupting the PI3K/Akt/mTOR signalling cascade [35]. The PI3K/Akt/mTOR axis, which is involved in cancer cell proliferation, differentiation and cellular metabolism, is frequently activated in many cancers and is one of the most significant molecular pathways in mRCC [36, 37]. Activation of the PI3K/Akt/mTOR pathway is correlated with aggressive behaviour and poor prognosis of RCC tumours and is more significantly altered in ccRCC, high TNM stage tumours, and tumours with poor prognostic features [38, 39]. SPOP binding to the substrate is a crucial event for E3 ligase-mediated ubiquitination and subsequent proteasome degradation. Levels of the PI3K/Akt pathway have been found to be correlated with SPOP expression, which could inhibit colorectal cancer and osteosarcoma invasion by significantly reducing the levels of PI3K and p-Akt [40, 41]. In the present study, there was a significant difference in the sensitivity of different cell lines overexpressing SPOP to several concentrations of sunitinib. It could be a promising potential molecular mechanism that may provide an effective therapeutic strategy for patients with advanced kidney cancer by exploring the relationships among the SPOP, VEGF and PI3K/Akt/mTOR pathways.

In addition, the Hedgehog signalling pathway, which increases tumour invasion and metastatic potential, is another important molecular mechanism that is worth investigating in the future. Aberrant activation of the Hedgehog pathway is associated with tumorigenesis in some cancers, including RCC, and plays an important role in RCC development [42–44]. Limited studies suggest that SPOP suppresses tumour development by negatively regulating the Hedgehog/Gli2 signalling pathway in gastric cancer [17]. In addition, the expression levels of the Hedgehog signalling pathway component genes Gli1 and Gli2, which are activated by the PI3K/Akt signalling pathway in RCC, are significantly elevated in ccRCC and provide a promising therapeutic strategy for RCC [45]. Currently, the Hedgehog inhibitors (HHIs) vismodegib and sonidegib are approved for use in advanced BCC, and other potential uses for the treatment of solid tumours beyond BCC are under development or in clinical trials [46]. Given the above, SPOP, as a tumour suppressor protein, plays an important role in inhibiting tumorigenesis by regulating different signalling pathways. However, studies on the molecular mechanism of the SPOP protein in RCC are still limited. Exploring the underlying mechanisms of signalling pathways in kidney cancer in detail is the best approach to provide a theoretical basis for the development of novel therapeutic strategies for mRCC patients in the future.

Differential expression levels or mutation profiles of SPOP in tumours play different roles in tumorigenesis and cancer progression [13, 14, 16]. Several studies have shown that SPOP expression is downregulated in some primary tumours, including gastric cancer, liver cancer, colorectal cancer, pancreatic cancer and non-small cell lung cancer, and low expression of SPOP is associated with poor prognosis in patients [17, 29–31, 47]. In the present study, immunohistochemical staining demonstrated that the SPOP protein was mainly expressed at low levels in the cytoplasm of ccRCC tissues and was relatively highly expressed in most adjacent normal tissues. The inhibitory role of SPOP was confirmed by an earlier study that showed that downregulation of SPOP expression in cancers might inhibit its functions as a tumour suppressor gene and promote cancer development [48]. Based on the immunohistochemical staining and cell culture experiment results in the present study, SPOP may act as a potential tumour suppressor protein in RCC tumorigenesis. However, the findings of the survival analysis did not provide supportive evidence showing a correlation of SPOP expression and overall survival, suggesting that high expression of SPOP could not be regarded as a hallmark of RCC and could not yet predict the prognosis of patients. The exact role of the SPOP protein in RCC is controversial and still needs to be confirmed by further research using a large cohort of samples.

As some studies showed that the tumor-promoting and tumor-suppressing activities of SPOP may be owing to differential subcellular localization of SPOP or differential expression of SPOP substrates in the cell and cancer typespartially to alter its substrate availability. If the substrates that bind to SPOP have tumor-suppressor roles, SPOP overexpression can play a tumor promoting role. Similarly, SPOP can have a tumor-suppressing role if the majority of substrates that bind to SPOP play the tumor promoting role [14]. For example, previous studies have shown that SPOP is differentially detected in gastric cancer tissues and adjacent gastric tissues that SPOP was overexpressed in the cytoplasm and nuclear of adjacent gastric mucosa epithelium cells but rarely expressed in gastric cancer cells.Further study provide evidence that SPOP functions as a tumor suppressor in different gastric cancer cell lines and through inhibiting Hh/Gli2 pathway, and the possible molecular mechanism of Gli2 stability regulated by SPOP [17]. Hence, though SPOP was mainly expressed in adjacent normal tissue cells but less staining in RCC cancer cells,the role of SPOP expression in cancer development is context and substrates dependent. So, it is important for us to pay attention to the potential substrate molecules of SPOP protein in RCC in future studies.Moreover, the mechanism and other role of differential expression of SPOP in RCC and paracancer tissues remain to be further elucidated. Molecular alterations in benign renal tissue in RCC may already have a premalignant potential interfering with local recurrence according to the previous studies [49–51]. Tumor-adjacent renal tissue may have oncological relevance,that the tumor progression or local recurrence may be associated with the molecular status of the tumor adjacent tissue [52, 53]. The tumor microenvironment (TME), including immune cells,adjacent normal cells and so on plays a crucial role in influencing tumor behavior and progression [54]. Some interesting yet complex events occur at tumor borders that contribute to the aberrant molecular changes occurring in adjacent renal tissue. For example, early phase RCC could mobilize endothelial progenitor cells (EPCs) into tumor-adjacent tissues, that the process of RCC invasion was promoted through synthetizing the stromal cell-derived factor-1 (SDF-1) and vascular endothelial growth factor (VEGF) [55]. So, further molecular studies to clarify the differences of SPOP expression and understanding the molecular events in RCC and adjacent renal tissue provide a possible contribution to understanding therapeutic resistance and designing new therapeutic agents.

Although there are some important discoveries in the present study, some limitations need to be discussed. First, the concentration gradient of drug experiments was too large to accurately reflect the significant concentration. Second, it is widely accepted that RCC is a heterogeneous tumour with distinct pathological tissue subtypes, including clear cell, papillary, and chromophobe subtypes. The TMA tissues used in this study consisted of a single pathological tissue subtype and could not be used to explore the expression of SPOP in the different RCC subtypes. More pathological tissue subtypes should be included to analyse the expression of SPOP in RCC tissue in the future, especially fresh frozen tissue from RCC radical nephrectomy.

## Conclusions

Briefly, we report that SPOP reduces tumorigenesis features in RCC cell lines and induces cell apoptosis in vitro. In human RCC samples, SPOP is expressed at low levels in the majority of ccRCC samples and at higher levels in most adjacent nontumor samples. All of these findings suggest that SPOP may act as a potential tumour suppressor protein in the tumorigenesis of human RCC. Further studies with a larger patient cohort and analysis of molecular mechanisms are needed to confirm our findings.

## Supplementary Information

**Additional file 1.** Supplementary Information file.

## Data Availability

The datasets used and/or analyzed during the current study are available from the corresponding author on reasonable request.

## Abbreviations

SPOP: Speckle-type POZ protein
RCC: Renal cell carcinoma
TMA: Tissue microarray
ccRCC: Clear cell renal cell carcinoma
mRCC: Metastatic renal cell carcinoma
CRL3: Cullin3-RING ubiquitin ligase
AR: Androgen receptor
SRC-3: Steroid receptor coactivator-3
PTEN: Phosphatase and tensin homolog deleted on chromosome ten
AR: Androgen Receptor
DMEM: Dulbecco’s modified Eagle’s medium
FBS: Foetal bovine serum
VEGF: Vascular endothelial growth factor
HHIs: Hedgehog inhibitors
SDF-1: Stromal cell derived factor-1
EPCs: Endothelial progenitor cells
BCC: Basal cell carcinoma
