# Supplementary material for: Speckle-type POZ protein could play a potential inhibitory role in human renal cell carcinoma
Source: BMC Cancer. 2022 Dec 7;22:1277. doi: 10.1186/s12885-022-10340-w (PMC9727862; doi:10.1186/s12885-022-10340-w)

**Supplementary Information file**


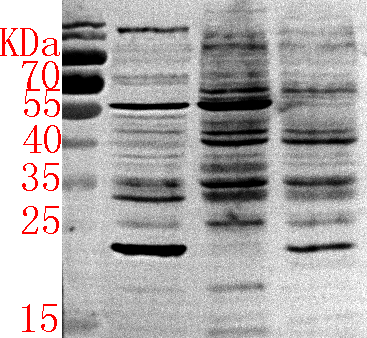
1.SPOP expression in cells before transfection(SPOP-42KDa)

caki-1 caki-2 ACHN


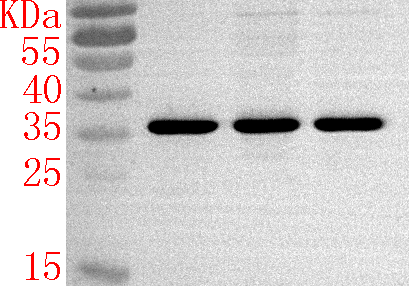
2.GAPDH-36KDa


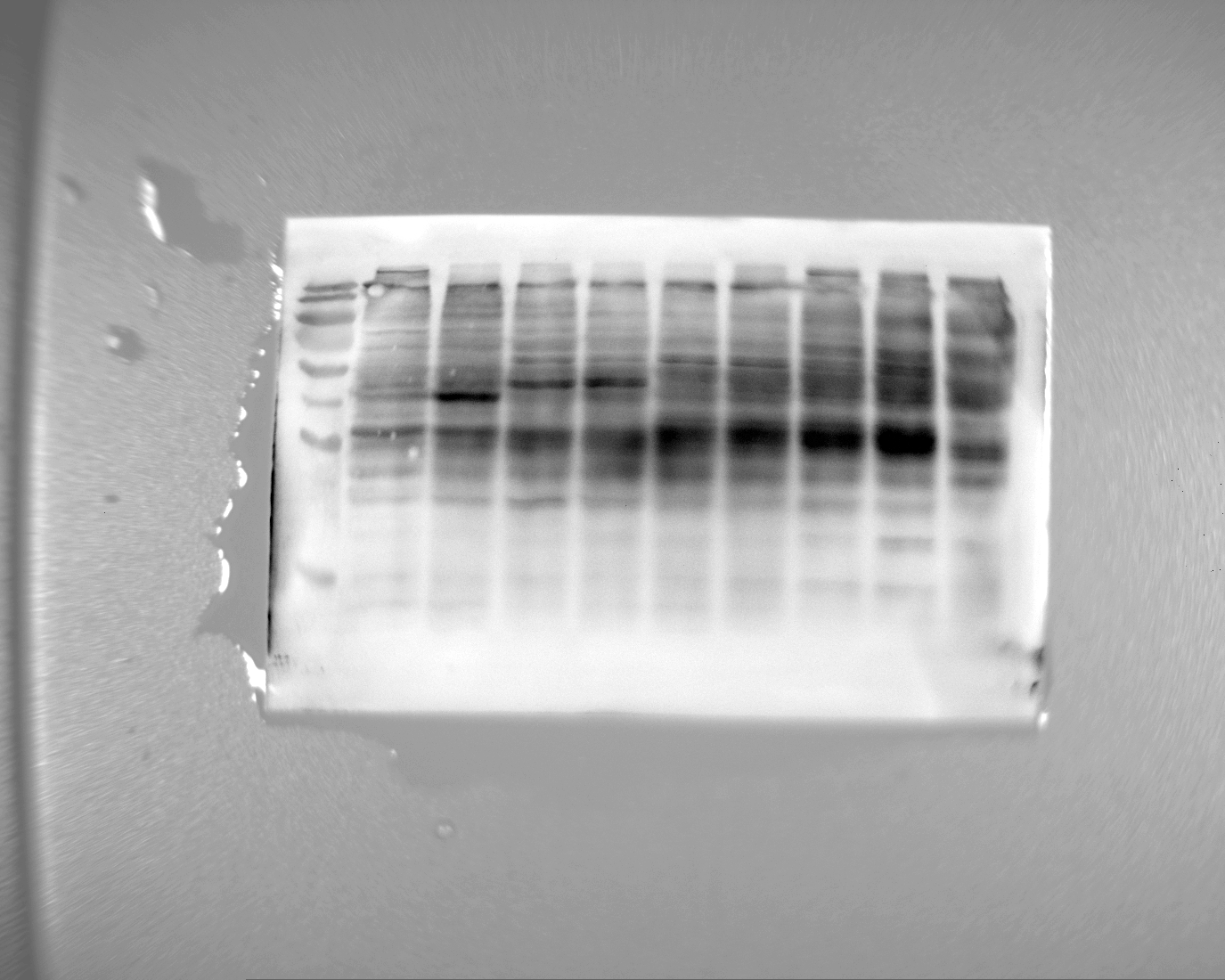
3.SPOP expression in cells after transfection(SPOP-42KDa)


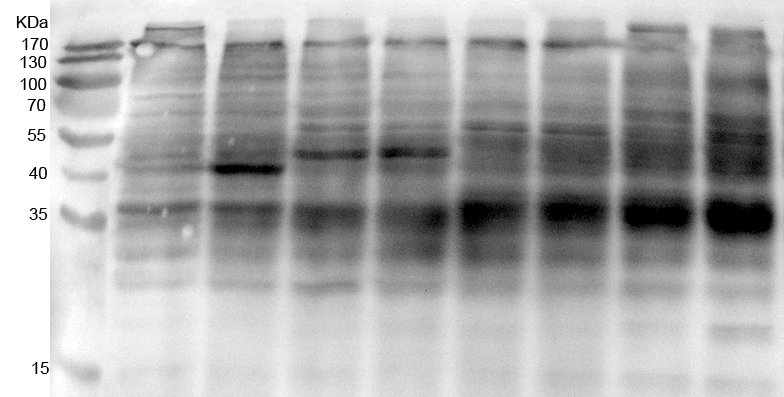


spop-ACHN spop-caki-1


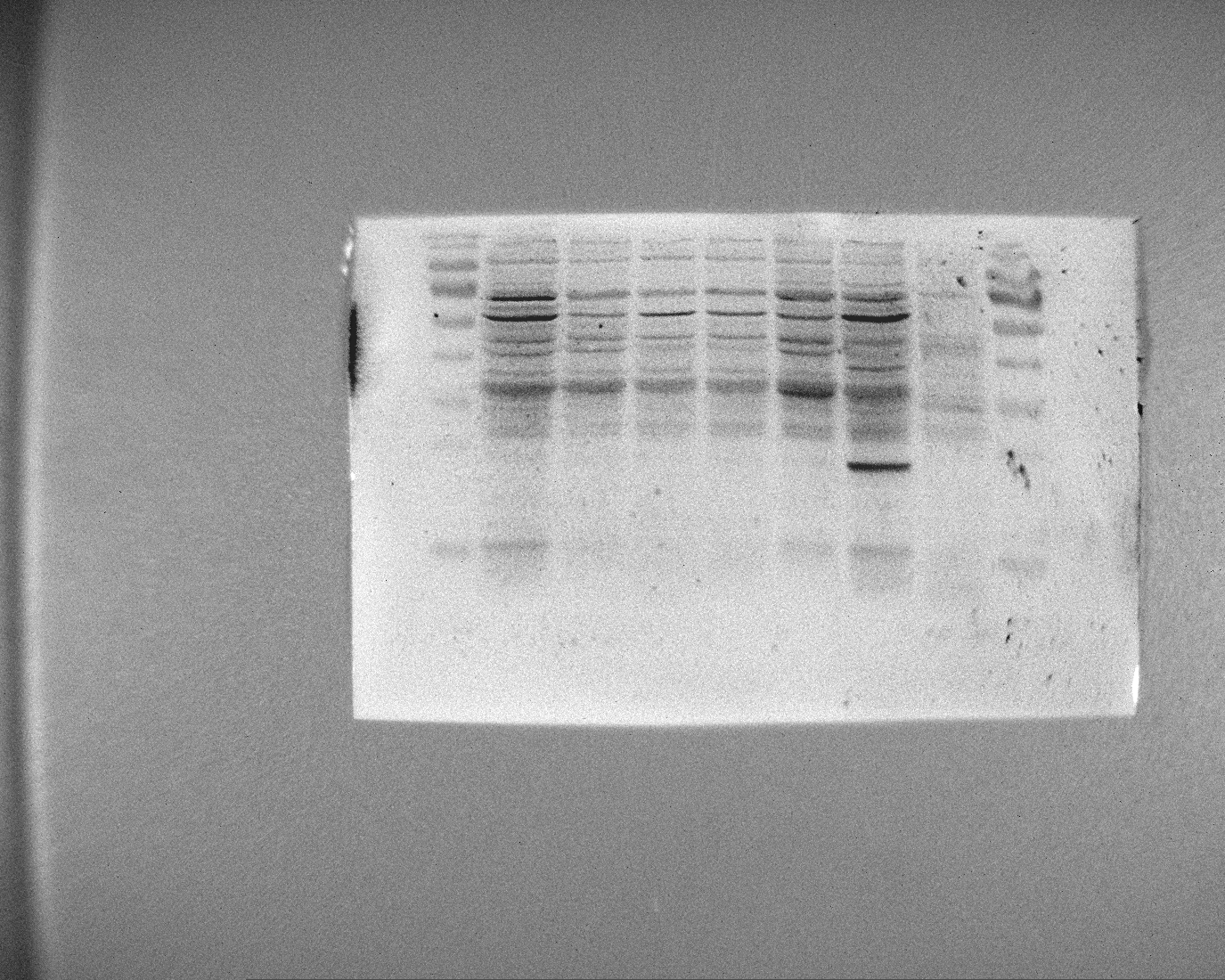
4.Caki-2 after silence


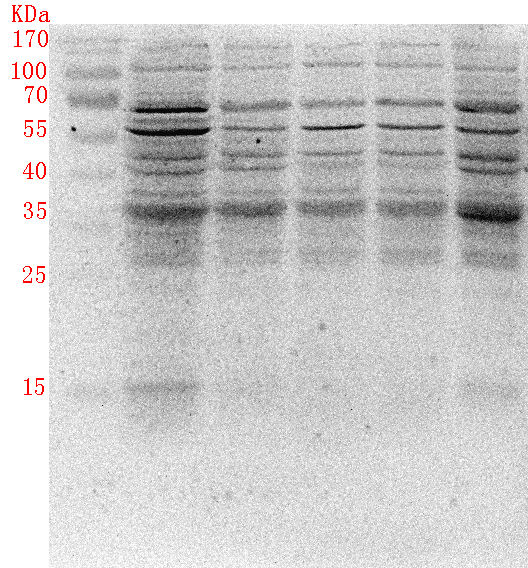


caki2-sh-NC caki2-sh-spop


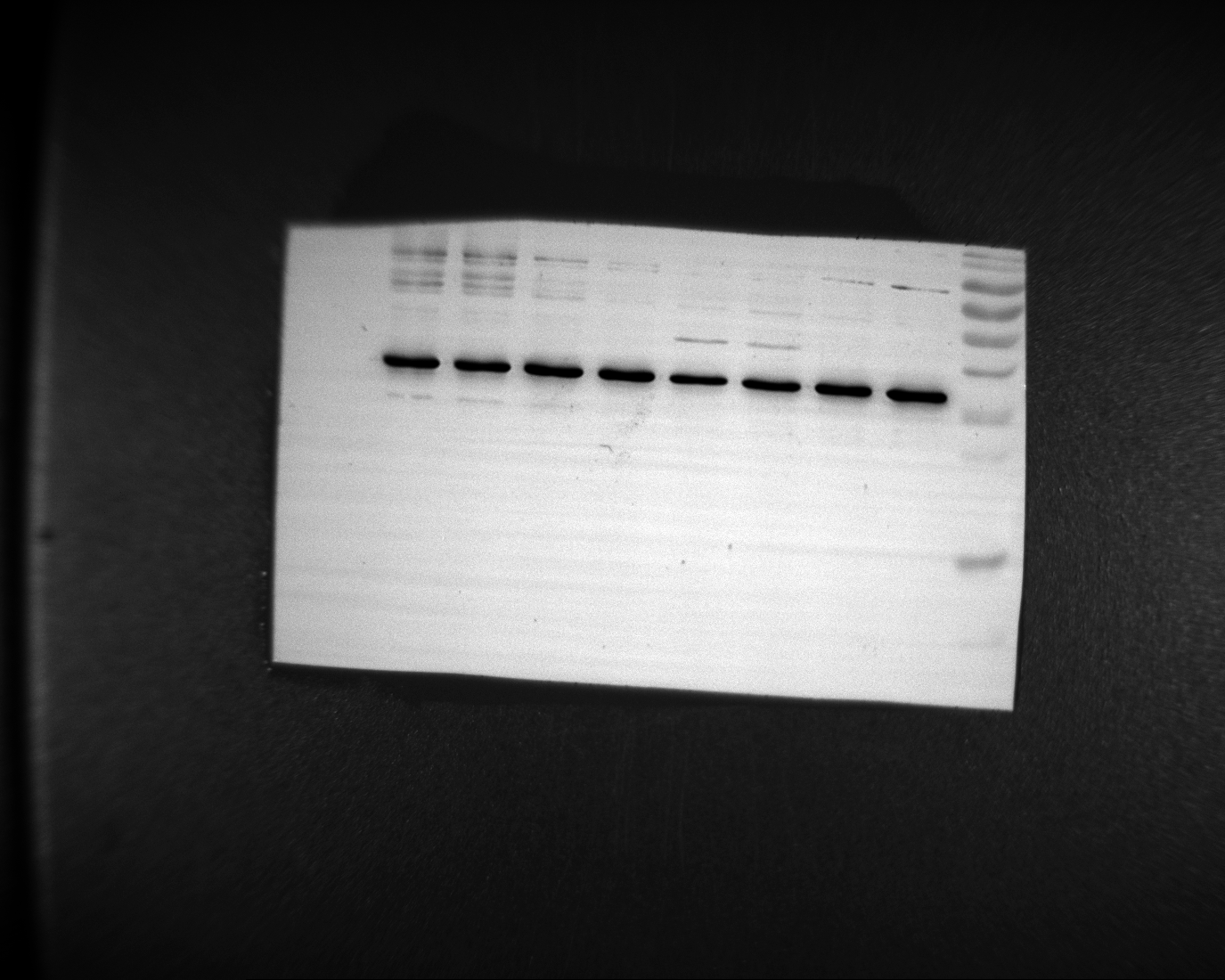
5.GAPDH-36KDa


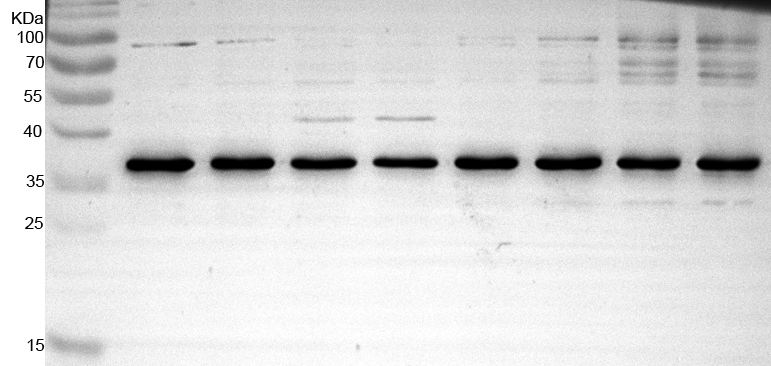


1. qPCR analyses


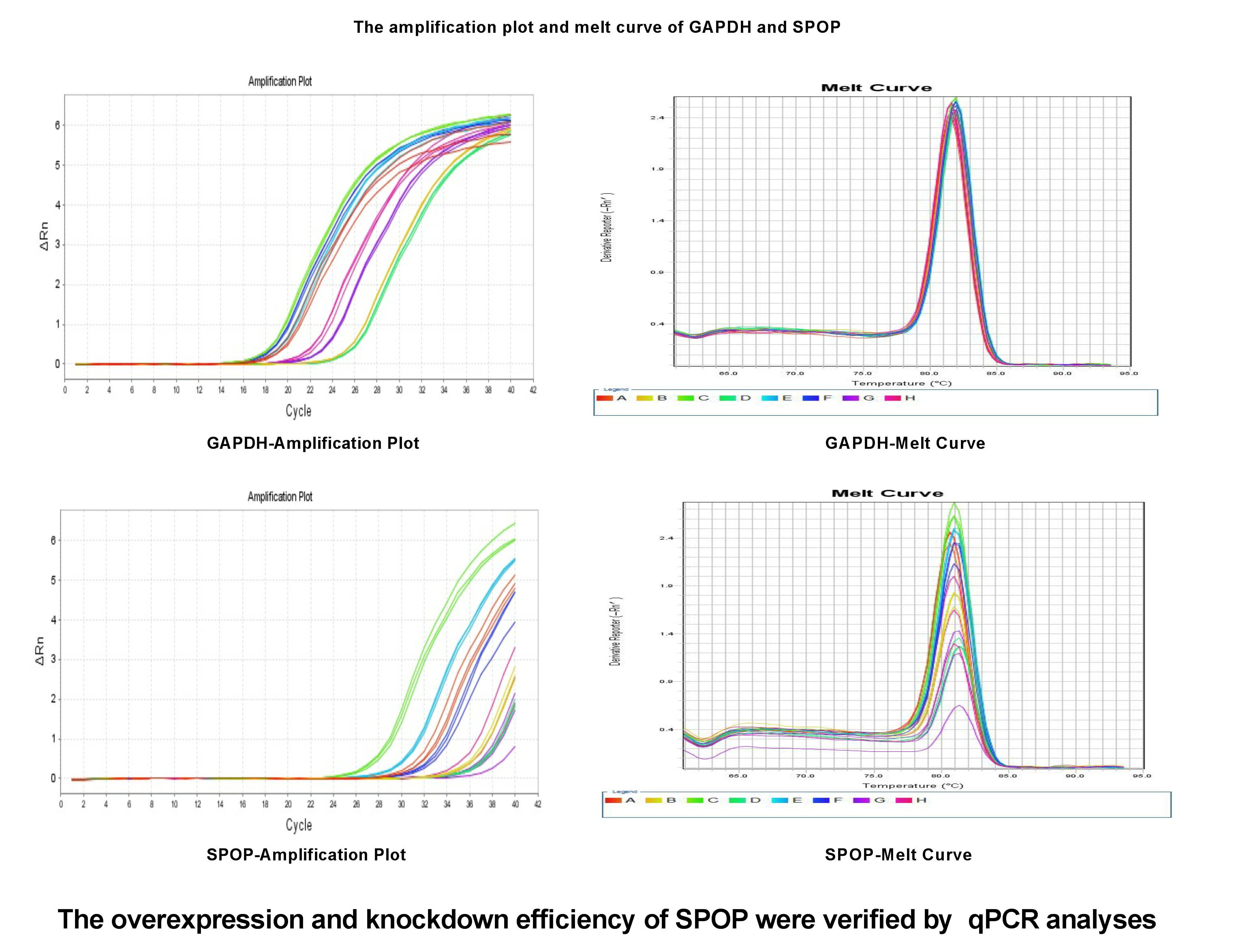

Supplement: Supplementary file 1 — Additional file 1. Supplementary Information file. [file 12885_2022_10340_MOESM1_ESM.docx]
